# Synthesis of some NH- and NH,S- substituted 1,4-quinones

**DOI:** 10.3906/kim-2011-3

**Published:** 2021-04-28

**Authors:** Ayşecik KAÇMAZ

**Affiliations:** 1 Department of Chemistry, Faculty of Engineering, İstanbul University-Cerrahpaşa, İstanbul Turkey

**Keywords:** Quinones, amines, *p*-chloranil, *p*-toluquinone, 2,3-dichloro-1, 4-naphthoquinone

## Abstract

A series of NH-substituted-1,4-quinones, possessing one, two, three or not chlorine, were synthesized by the reaction between different quinones (*p*-chloranil (**1**), *p*-toluquinone (**2**), or 2,3-dichloro-1,4-naphthoquinone (**3**)) and (-)-*cis*-myrtanylamine (**5**) via nucleophilic reactions. Moreover, 2-bromo-1,4-naphthoquinone (**4**) was reacted with 2-(methylthio)ethylamine (**11**) to produce amino-substituted naphthoquinones (**12** and **13**), bearing with bromine and not bromine. In addition, 2-bromo-1,4-naphthoquinone (**4**) was reacted with 4′-aminodibenzo-18-crown-6 (**14**) and 4′-aminobenzo-18-crown-6 (**16**) to yield crown-containing 1,4-naphthoquinones (**15** and **17**), respectively. New compounds were characterized, providing ^1^H NMR, ^13^C NMR, FTIR, MS-ESI, UV/Vis and elemental analysis.

## 1. Introduction

Quinones are widespread in nature [1,2] (in plants, fungi, bacteria etc.), and many synthetic or natural quinones possess various pharmacological properties including anticancer [3–5], antibacterial [6], antifungal [6], antiinflammatory [7], antimycobacterial [8], and molluscicidal [9] activities. Moreover, substituents such as halogen, amino, thio groups of the synthetic quinone derivatives can increase their pharmacological activities, such as antibacterial, cytotoxic, and antiproliferative [3,10,11]. Quinonoid systems’ pharmacological specialties are related to their capacity to produce free radicals or semiquinones in redox reactions [11–13].

Among quinones, 1,4-naphthoquinone scaffold are found in many natural or synthetic products such as menadione, juglone, plumbagin, alkannin, and shikonin [14–16]. In addition, 1,4-naphthoquinone derivatives have receieved a considerable interest in biological applications with their antibacterial [11], antiatherosclerosis [17], antiinflammatory [18], anticancer [5,18], and cytotoxic [19] activities. Thus, many reports on the reactions of 1,4-naphthoquinones with amines [3,5,13], anilines [11,20], phenols [21], thiols [3,5,22], aminopyridine [23], alcohol [24,25], glycol [25] are available in the literature. In this study, compounds 10, 12, 13, 15, 17, and 19, 20 have NH- and NH,SR- substituted-1,4-naphthoquinone skeleton, respectively. 

The literature mentions some aminobenzocrown ethers [26,27] similar to 15 and 17. For example, N-(2-chloro-1,4-naphthoquinon-3-yl)-4’-aminobenzocrown ethers were synthesized from the reaction between 4’-aminobenzocrown ethers and 2,3-dichloro-1,4-naphthoquinone [26], and thus in the present study, synthesis of 15 and 17 contribute to crown-containing naphthoquinones, carrying out the reaction between crown ethers (14 and 16) and 2-bromo-1,4-naphthoquinone (4). Moreover, crown-containing naphthoquinone 19 was synthesized, including both amino and thio substituents, together. 

1,4-benzoquinones, including NH-, methoxy, thio, alkyl or aryl groups, have been the subject of study due to their properties such as antimicrobial [28], antibacterial [29], cytotoxic [30–32], potential urease inhibitor [33], and potent inhibitory activity towards enzyme system [34]. In addition, some of studies on the formation of N(H)-, SR-, alkoxy substituted-1,4-benzoquinones have been reported in the literature [34–38]. Among 1,4-benzoquinones, halogenanils, such as chloranil, with amines yield amination products of quinones. For example, Wu H et al. reported tetrathiafulvalene-quinone dyad, having mono-NH-substituted-tri-chloro-1,4-benzoquinone structure [38]. In another example, Sing and et al. synthesized [39] new coordination polymers, starting from 2,5-dichloro-3,6-bis(ethylamino)-1,4-benzoquinone. In this study, compounds 6 and 7 have mono-NH-substituted-tri-chloro-1,4-benzoquinone and 2,5-dichloro-3,6-bis(NH-substituted)-1,4-benzoquinone structures, respectively, synthesized from p-chloranil 1 and primary amine 5. Moreover, compounds 8 and 9 are di-amination products of methyl-p-benzoquinone 2. 

Different research groups from our university have reported some N-, NH- or SR- substituted 1,4-naphtho(benzo) quinones [22,40–45]. Some of these compounds have antifungal, antibacterial, antioxidant, and cytotoxic activities. Recently, our research group have reported some 1,4-quinone derivatives [46–49] with their antifungal, antibacterial activities, electrochemical properties, or antiproliferative effects. Moreover, in the literature, there are many reports regarding biologically important compounds, including benzoquinone or naphthoquinone core [3,30,50].

The importance of this kind of compounds has motivated this study to synthesize 1,4-naphtho(benzo)quinones bearing with amino and/or thio. Thus, *p-*chloranil 1, methyl-p-benzoquinone 2, dichloro-1,4-naphthoquinone 3 and 2-bromo-1,4-naphthoquinone 4 were used as lead molecules, as shown Figure. Various spectroscopic techniques (UV/Vis, FTIR, ^1^H NMR, ^13^C NMR, MS-ESI) have been employed to characterize the synthesized compounds. It is expected that the new synthesized compounds will be useful for pharmacological field with their potential biological activities. 

**Figure:**
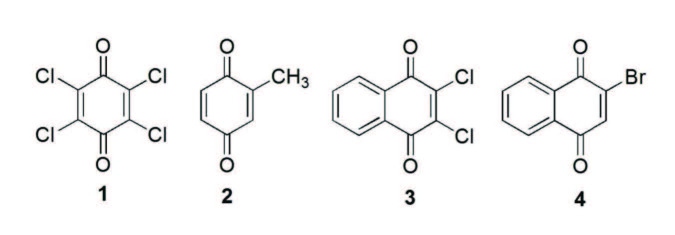
Quinones used in the present work (p-chloranil 1, methyl-p-benzoquinone 2, 2,3-dichloro-1,4-naphthoquinone 3 and 2-bromo-1,4-naphthoquinone 4).

## 2. Materials and methods

### 2.1. Chemistry

All the chemicals used (1, 2, 3, 4, 5, 11, 14, 16, 18) were commercially purchased and used without further purification. To measure melting points, Buchi B-540 was used. The elemental analyses, IR spectra, and UV-Vis spectra were carried out by using the ThermoFinnigan Flash EA1112, Thermo Scientific Nicolet 6700, and Shimadzu UV/Vis spectrophotometer 2600 (in CHCl_3_), respectively. The UV-Vis spectra were recorded on a Shimadzu UV/Vis spectrophotometer 2600, in CHCl_3_. The mass spectra were performed on a ThermoFinnigan LCQ AdvantageMAX system. ^1^H and ^13^C NMR spectra were performed in CDCl_3_ solution on a spectrometer (Varian Unity Inova). Chemical shifts (δ, ppm) are reported by using tetramethylsilane as internal standard. Column chromatography was performed on glass columns by using silica gel (70–230 mesh). 

### 2.2. Synthesis of quinonoid compounds

#### Synthesis of 2-((6,6-dimethylbicyclo[3.1.1]heptan-2-ylmethyl)amino)-3,5,6-trichlorocyclohexa-2,5-diene-1,4-dione (6) and 2,5-bis((6,6-dimethylbicyclo[3.1.1]heptan-2-ylmethyl)amino)-3,6-dichlorocyclohexa-2,5-diene-1,4-dione (7)

The solution of 1 (640 mg, 2.6 mmol) and (-)-cis-myrtanylamine 5 (400 mg, 2.6 mmol) in dichloromethane was allowed to stir at room temperature by monitoring the progression of the reaction mixture with Thin-layer chromatography (TLC). Then, the reaction mixture was extracted with water and CHCl_3_. The organics were dried over sodium sulfate and removed under vacuo; thus, the crude mixture was obtained. The crude mixture was then purified by column chromatography on silica gel (stationary phase) with n-hexane/CH_2_Cl_2_ (1/2) (mobil phase) to afford products 6 and 7. 

**2-((6,6-dimethylbicyclo[3.1.1]heptan-2-ylmethyl)amino)-3,5,6-trichlorocyclohexa-2,5-diene-1,4-dione (6):** R_f_ = 0.8 (CH_2_Cl_2_); Yield: 10% (100 mg); Dark purple viscous oil; UV (CHCl_3_), λ_max_, nm (log ε): 244 (4.87), 320 (4.71), 529 (4.06); IR (ATR): 3336, 2906, 2870, 1683, 1648, 1606, 1572, 1514, 1459, 1218, 1083; ^1^H NMR (CDCl_3_) δ: 5.88 (^1^H, NH, brs), 3.60–3.80 (m, 2H, -CH_2_-NH), 2.20-2.40 (m, 2H), 1.80–2.00 (m, 5H), 0.60–1.40 (m, 8H); ^13^C NMR (CDCl_3_) δ: 174.48 (C=O), 173.15, 143.09, 135.59, 129.40, 95.81, 50.63, 43.48, 42.62, 41.23, 38.72, 33.23, 29.71, 27.90, 25.82, 23.22, 19.60; MS m/z 360.4 ([M-H]^-^, 100%). Anal. calc. for C_16_H_18_Cl_3_NO_2_ (362.68): C 52.99, H 5.00, N 3.86; Found: C 53.25, H 5.10, N 3.98.

**2,5-bis((6,6-dimethylbicyclo[3.1.1]heptan-2-ylmethyl)amino)-3,6-dichlorocyclohexa-2,5-diene-1,4-dione (7): **R_f_ = 0.9 (CH_2_Cl_2_); Yield: 36% (225 mg); Grey solid; m.p. 233–235 °C; UV (CHCl_3_), λ_max_, nm (log ε): 361 (4.92), 242 (4.25); IR (ATR): 3244, 2897, 1655, 1567, 1489, 1440, 1330, 1056; ^1^H NMR (CDCl_3_) δ: 7.18 (brs, 2H, NH), 3.88–3.96 (2H, m), 3.75–3.87 (2H, m), 2.30–2.50 (4H, m), 1.80–2.10 (10H, m), 1.40–1.60 (m, 2H), 1.21 (s, 6H), 1.04 (s, 6H), 0.95 (d, 2H, ^3^J = 9.75 Hz); ^13^C NMR (CDCl_3_) δ: 172.12 (C=O), 145.44 (C-N), 99.23 (C-Cl), 50.20, 43.44, 42.56, 41.23, 38.67, 33.21, 33.17, 27.87, 27.84, 25.84, 23.20, 23.19, 19.52; MS m/z 479.1 ([M+H]^+^, 100%). Anal. calc. for C_26_H_36_Cl_2_N_2_O_2_ (479.48): C 65.13, H 7.57, N 5.84; Found: C 65.43, H 7.85, N 5.99.

#### Synthesis of 3,5-bis((6,6-dimethylbicyclo[3.1.1]heptan-2-ylmethyl)amino)-2-methylcyclohexa-2,5-diene-1,4-dione (8) and 2,5-bis((6,6-dimethylbicyclo[3.1.1]heptan-2-ylmethyl)amino)cyclohexa-2,5-diene-1,4-dione (9)

The solution of methyl-*p-*benzoquinone 2 (398 mg, 3.26 mmol) and (-)-cis-myrtanylamine 5 (500 mg, 3.26 mmol) in EtOH (20 mL) and water (1.5 mL) in the presence of Na2CO3 was allowed to stir at room temperature by monitoring the progression of the reaction mixture with TLC. Then, the reaction mixture was extracted with water and CHCl_3_. The organics were dried over sodium sulfate and removed under vacuo; thus, the crude mixture was obtained. The crude mixture was then purified by column chromatography on silica gel (stationary phase) with n-hexane/CH_2_Cl_2_ (2/1) (mobil phase) to afford products 8 and 9.

**3,5-bis((6,6-dimethylbicyclo[3.1.1]heptan-2-ylmethyl)amino)-2-methylcyclohexa-2,5-diene-1,4-dione (8):** Rf= 0.6 (CH_2_Cl_2_); Yield: 10% (69 mg); Purple solid; m.p. 198–200 °C; IR (cm^-1^): ν = 3250, 2898, 1637, 1601, 1553, 1458, 1341, 1237, 1088; ^1^H NMR (CDCl_3_) δ: 6.72 (brs, 2H, NH), 5.25 (s, ^1^H, CH_quinone_), 3.59 (d, 2H, CH_myrt_, ^3^J = 7.80 Hz), 3.07–3.19 (m, 2H, CH_myrt_), 2.25–2.45 (m, 4H, CH_myrt_), 2.07 (s, 3H, CH_3quinone_), 1.82–2.05 (10H, m, CH_myrt_), 1.41–1.56 (2H, m, CH_myrt_), 1.21 (d, 6H, CH_myrt_, J = 4.39 Hz), 1.03 (d, 6H, CH_myrt_, J = 3.90 Hz), 0.93 (t, 2H, CH_myrt_, J = 7.81 Hz); ^13^C NMR (CDCl_3_) δ: 178.98, 178.88 (C=O), 150.91, 148.13, 101.77, 91.71, 50.54, 48.25, 43.77, 43.52, 42.55, 41.27, 41.20, 40.24, 38.69, 38.64, 33.27, 33.14, 27.91, 27.88, 27.85, 25.88, 23.26, 23.23, 19.94, 19.74; 10.44 (CH_3quinone_); MS m/z 425.3 ([M+H]^+^, 100%). Anal. calc. for C_27_H_40_N_2_O_2_ (424.62): C, 76.37; H, 9.50; N, 6.60; Found C, 75.97; H, 9.49; N, 6.69. 

**2,5-bis((6,6-dimethylbicyclo[3.1.1]heptan-2-ylmethyl)amino)cyclohexa-2,5-diene-1,4-dione (9):**^[47]^ Light pink solid; m.p 281–282 °C; (lit[47]: 280–282 °C); Yield: 78% (524 mg); ^1^H NMR (CDCl_3_) δ: 6.61 (2H, 2×NH, brs), 5.23 (2H, 2×CH_quinone_), 3.02–3.14 (m, 4H, 2 -CH_2_-NH), 2.26–2.36 (m, 4H); 1.78–1.98 (m, 10H); 1.36–1.46 (m, 2H), 1.13 (s, 6H, 2×CH_3_), 0.96 (s, 6H, 2×CH_3_), 0.86 (s, H), 0.84 (s, H); ^13^C NMR (CDCl_3_) δ: 177.02 (C=O); 150.48 (=C-NH-); 91.62, 91.57 (C-H_quin_); 47.31, 47.18, 42.79, 40.23, 39.27, 37.66, 32.14, 26.87, 24.87, 22.24, 18.98. MS m/z 411.3 ([M+H]^+^, 100%).

#### Synthesis of 2-((6,6-dimethylbicyclo[3.1.1]heptan-2-ylmethyl)amino)-3-chloronaphthalene-1,4-dione (10)

The solution of 2,3-dichloro-1,4-naphthoquinone 3 (740 mg, 3.26 mmol) and (-)-cis-myrtanylamine 5 (500 mg, 3.26 mmol) in CH_2_Cl_2_ was allowed to stir at room temperature by monitoring the progression of the reaction mixture with TLC. Then, the reaction mixture was extracted with water and CHCl_3_. The organics were dried over sodium sulfate and removed under vacuo; thus, the crude mixture was obtained. The crude mixture was then purified by column chromatography on silica gel (stationary phase) with n-Hexane/CH_2_Cl_2_ (1/3) (mobil phase) to afford product 10: R_f_ = 0.6(CH_2_Cl_2_); Yield: 40% (450 mg); Red solid; m.p. 179–181 °C; UV (CHCl_3_), λ_max_, (log ε): 278 (4.48), 474 (3.58); IR (ATR): 3267, 2978, 2908, 1680, 1596, 1554, 1514, 1408, 1294, 1252, 1062; ^1^H NMR (CDCl_3_) δ: 8.07 (dd, H, CH_napht_, ^3^J = 7.3 Hz, ^4^J = 1.0 Hz); 7.95 (dd, H, CH_napht_, ^3^J = 7.8 Hz, ^4^J = 1.0 Hz); 7.64 (td, H, CH_napht_, ^3^J = 7.6 Hz, ^4^J = 1.1 Hz); 7.53 (td, H, CH_napht_, ^3^J = 7.3 Hz, ^4^J = 1.1 Hz); 6.03 (^1^H, NH, brs); 3.70–3.90 (m, 2H, -CH_2_-NH-); 2.20–2.40 (m, 2H); 1.80–2.00 (m, 4H); 1.40–1.60 (m, H); 1.14 (s, 3H); 0.98 (s, 3H); 0.87 (d, H, ^3^J = 9.76 Hz); ^13^C NMR (CDCl_3_) δ: 180.5, 176.7 (C=O_napht_); 144.3(=C-N); 134.9, 132.8, 132.3, 129.7, 126.8 (C_napht_, C-H_napht_); 50.3, 43.5, 42.7, 41.3, 38.7, 33.3, 27.9, 25.9, 23.2, 19.7; MS m/z 342.5 ([M]^-^, 100%). Anal. calc. for C_20_H_22_ClNO_2_ (343.85): C 69.86, H 6.45, N 4.07; Found: C 69.47, H 6.55, N, 3.75. 

#### 2.2.4. Synthesis of 2-(2-(methylthio)ethylamino)-3-bromonaphthalene-1,4-dione (12) and 2-(2-(methylthio)ethylamino)naphthalene-1,4-dione (13)

A solution of 4 (1.3 g, 5.48 mmol) and 2-(methylthio)ethylamine 11 (0.5 g, 5.48 mmol) in CH_2_Cl_2_ was allowed to stir at room temperature by monitoring the progression of the reaction mixture with TLC. Then, the reaction mixture was extracted with water and CHCl_3_. The organics were dried over sodium sulfate and removed under vacuo; thus, the crude mixture was obtained. The crude mixture was then purified by column chromatography on silica gel (stationary phase) with n-Hexane/CH_2_Cl_2_ (1/1) (mobil phase) to afford products 12 and 13.

**2-(2-(methylthio)ethylamino)-3-bromonaphthalene-1,4-dione (12):**R_f_ = 0.5(CH_2_Cl_2_); Yield: 7% (125 mg); Dark red solid; m.p. 102–104 °C; UV (CHCl_3_), λ_max_, nm (log ε): 277 (4.48), 487 (3.42); IR (ATR): 3306, 1673, 1591, 1560, 1513, 1441,1327, 1251, 1123; ^1^H NMR (CDCl_3_) δ: 8.12 (d, ^1^H, CH_napht_, ^3^J = 7.3 Hz); 8.02 (d, ^1^H, CH_napht_, ^3^J = 7.7 Hz), 7.71 (t, ^1^H, CH_napht_, ^3^J = 7.6 Hz), 7.62 (t, ^1^H, CH_napht_, ^3^J = 7.6 Hz), 6.44 (brs, ^1^H, NH), 4.08 (t, 2H, NH-CH_2_, ^3^J = 6.3 Hz); 2.83 (t, 2H, CH_2_-S, ^3^J = 6.3 Hz); 2.16 (s, 3H, CH_3_); ^13^C NMR (CDCl_3_) δ: 179.97, 176.37 (C=O); 146.48, 134.79, 132.47, 132.44, 132.22, 129.88, 127.03, 126.87 (C_napht_, CH_napht_); 43.00 (NH-CH_2_), 34.68 (CH_2_-S), 15.06 (CH_3_); MS m/z 324.0 ([M-H]^-^, 100%). Anal. calc. for C_13_H_12_BrNO_2_S (326.21): C 47.86, H 3.71, N 4.29; Found: C 48.28, H 3.64, N 4.04.

**2-(2-(methylthio)ethylamino)naphthalene-1,4-dione (13):**R_f_ = 0.2(CH_2_Cl_2_); Yield: 30% (410 mg); Orange solide; m.p 139–141 °C; UV (CHCl_3_), λ_max_ (log ε): 271 (4.21), 442 (3.37); IR (ATR): 3360, 3237, 2910, 1664, 1591, 1563, 1498, 1444, 1339, 1227, 1068; ^1^H NMR (CDCl_3_) δ: 8.09 (dd, ^1^H, CH_napht_, ^3^J = 7.7 Hz, ^4^J = 1.0 Hz); 8.04 (dd, ^1^H, CH_napht_, ^3^J = 7.6 Hz, ^4^J = 1.1 Hz); 7.72 (td, ^1^H, CH_napht_, ^3^J = 7.6 Hz, ^4^J = 1.3 Hz); 7.61 (td, ^1^H, CH_napht_, ^3^J = 7.6 Hz, ^4^J = 1.3 Hz); 6.25 (brs, ^1^H, NH); 5.75 (s, ^1^H, CH_napht_); 3.40 (q, 2H, -NH-CH_2_, ^3^J = 6.3 Hz); 2.81 (t, 2H, -CH_2_-, ^3^J = 6.3 Hz); 2.15 (s, 3H, CH_3_); ^13^C NMR (CDCl_3_) δ: 183.0, 181.6 (C=O); 147.7; 134.7; 133.5;132.1; 130.5; 126.3; 126.2;101.2; 40.8 (NH-CH_2_); 32.2 (-CH_2_-S); 15.3 (CH_3_); MS m/z 247.9 ([M+H]^+^, 100%). Anal. calc. for C_13_H_13_NO_2_S (247.31): C 63.13, H 5.30, N 5.66, S 12.97; Found: C 63.12; H 5.12; N 5.58, S 13.33.

#### 2.2.5. Synthesis of Compound 15

The solution of 4 (63 mg, 0.27 mmol) and 4′-aminodibenzo-18-crown-6 14 (0.1 g, 0.27 mmol) with CH_3_COONa in CHCl_3_ and ethanol was allowed to stir at room temperature by monitoring the progression the reaction mixture with TLC. Then, the reaction mixture was extracted with water and CHCl_3_. The organics were dried over sodium sulfate and removed under vacuo; thus, the crude mixture was obtained. The crude mixture was then purified by column chromatography on silica gel (stationary phase) with ethyl acetate/CH_2_Cl_2_ (10/1) (mobil phase) to afford product 15: Yield: 69% (112 mg); Dark purple solid; m.p. 187–189 °C; IR (ATR): 3277, 2987, 2921, 1669, 1649, 1592, 1566, 1509, 1225; ^1^H NMR (CDCl_3_) δ: 8.21 (^1^H, dd, CH_napth_, ^3^J = 7.81 Hz, ^4^J =0.98 Hz), 8.13 (^1^H, dd, CH_napth_, ^3^J = 7.57 Hz, ^4^J = 1.21 Hz), 7.77 (td, ^1^H, CH_napth_, ^3^J = 7.54 Hz, ^4^J = 1.38 Hz), 7.70 (td, ^1^H, CH_napth_, ^3^J = 7.55 Hz, ^4^J = 1.31 Hz), 7.74 (brs, ^1^H, NH), 6.85–6.93 (m, 4H, CH_arom_), 6.82 (d, ^1^H, CH_arom_, ^3^J =8.49 Hz), 6.70 (dd,^1^H, CH_arom_, ^3^J = 8.43 Hz, ^4^J = 2.32 Hz), 6.66 (s, ^1^H, CH_arom_, ^4^J = 2.45 Hz), 4.13–4.25 (m, 8H, 4 x CH_2_), 4.00–4.10 (m, 8H, 4 x CH_2_); ^13^C NMR (CDCl_3_) δ: 180.11, 177.35 (C=O), 144.22, 134.99, 132.77, 132.54, 130.54, 129.74, 127.49, 127.08, 118.02, 113.18, 101.46; 69.88, 68.66 (CH_2_); MS m/z 608.3 ([M-H]^-^, 100%). Anal. calc. for C_30_H_28_BrNO_8_ (610.5): C, 59.03; H, 4.62; N, 2.29. Found C, 59.43; H, 4.92; N, 2.49.

#### 2.2.6. Synthesis of compound 17

The solution of 2-bromo-1,4-naphthoquinone 4 (72 mg, 0.30 mmol) and 4′-aminobenzo-18-crown-6 16 (0.1 g, 0.30 mmol) with Na2CO3 in CH_2_Cl_2_ was allowed to stir at reflux temperature by monitoring the progression of the reaction mixture with TLC. Then, the reaction mixture was extracted with water and CHCl_3_. The organics were dried over sodium sulfate and removed under vacuo; thus, the crude mixture was obtained. The crude mixture was then purified by column chromatography on silica gel (stationary phase) with ethyl acetate/CH_2_Cl_2_ (10/1) (mobil phase) to afford product 17: R_f_ = 0.8 (CH_3_OH); Yield: 29% (50 mg); Dark purple solid; m.p. 139–141 °C; IR (ATR): 3319, 2927, 2872, 1668, 1642, 1591, 1563, 1506, 1234, 1124; ^1^H NMR (CDCl_3_) δ: 8.13 (^1^H, dd, CH_napth_, ^3^J = 7.81 Hz, ^4^J =0.98 Hz), 8.04 (dd, ^1^H, CH_napth_, ^3^J = 7.81 Hz, ^4^J =0.98 Hz), 7.69 (td, ^1^H, CH_napth_, ^3^J = 7.57 Hz, ^4^J =1.46 Hz), 7.65 (brs, NH), 7.61 (td, ^1^H, CH_napth_, ^3^J = 7.57 Hz, ^4^J =1.46 Hz), 6.76 (d, ^1^H, CH_arom_, ^3^J = 8.29 Hz), 6.62 (dd, ^1^H, CH_arom_, ^3^J = 8.78 Hz, ^4^J =2.44 Hz), 6.59 (s, ^1^H, CH_arom_, ^4^J =2.44 Hz), 4.05–4.13 (m, 4H, 2 CH_2_), 3.82–3.90 (m, 4H, 2 CH_2_), 3.68–3.74 (m, 4H, 2 CH_2_), 3.62–3.68 (m, 8H, 4 CH_2_); ^13^C NMR (CDCl_3_) δ: 180.1, 177.3 (C=O), 148.5, 147.4, 144.2, 135.0, 132.8, 132.5, 130.7, 129.7, 127.5, 127.1, 127.0, 118.2, 111.9, 106.1, 70.8, 70.7, 70.6, 69.6, 69.4, 69.3, 69.1, 69.0; MS m/z 560.2 ([M-H]^-^, 100%). Anal. calc. for C_26_H_28_BrNO_8_ (562.41): C, 55.53; H, 5.02; N, 2.49. Found C, 55.13; H, 5.34; N, 2.77. 

#### 2.2.7. Synthesis of compound 19

The solution of 17 (40 mg, 0.07 mmol) and 1-dodecanethiol 18 (510 mg, 2.52 mmol) in CHCl_3_ in the presence of triethylamine (2-3 mL) was allowed to stir at reflux temperature by monitoring the progression of the reaction mixture with TLC. Then, the reaction mixture was extracted with water and CHCl_3_. The organics were dried over sodium sulfate and removed under vacuo;  thus, the crude mixture was obtained. The crude mixture was then purified by column chromatography on silica gel (stationary phase) with ethyl acetate/CH_2_Cl_2_ (1/1) (mobil phase) to afford product 19: Yield: 80% (39mg); Dark purple viscous oil; IR (ATR): 3444, 2957, 2921, 2851, 1665, 1591, 1550, 1513, 1230; ^1^H NMR (CDCl_3_) δ: 8.07 (^1^H, dd, CH_napth_, ^3^J = 7.81 Hz, ^4^J =0.98 Hz), 7.99 (dd, ^1^H, CH_napth_, ^3^J = 7.32 Hz, ^4^J =0.98 Hz), 7.74 (brs, ^1^H, NH), 7.68 (td, ^1^H, CH_napth_, ^3^J = 7.57 Hz, ^4^J =1.47 Hz), 7.59 (td, ^1^H, CH_napth_, ^3^J = 7.58 Hz, ^4^J =1.31 Hz), 6.71 (d, ^1^H, CH_arom_, ^3^J = 8.78 Hz), 6.52 (dd, ^1^H, CH_arom_, ^3^J = 8.30 Hz, ^4^J = 1.95 Hz), 6.49 (s, ^1^H, CH_arom_, ^4^J = 2.44 Hz), 4.00–4.15 (m, 4H, 2CH_2_crown), 3.80–3.95 (m, 4H, 2CH_2_crown), 3.68–3.80 (m, 4H, 2CH_2_crown), 3.60–3.68 (m, 8H, 4CH_2_crown), 3.21 (t, 2H, S-CH_2_-, ^3^J = 8.54 Hz), 1.65–0.85 (m, 20H, 10 CH_2_aliph), 0.80 (3H, t, CH_3_, ^3^J =7.08 Hz); ^13^C NMR (CDCl_3_) δ: 181.0, 180.4 (C=O); 147.5, 145.4, 145.3, 134.6, 133.6, 132.6, 132.4, 130.5, 126.8, 126.6, 126.5, 116.8, 115.4, 108.4 (CH_napht_, C_napht_, CH_arom_); 69.9, 69.7, 68.8, 67.7, 67.5, 58.9 (CH_2_crown); 31.9, 29.64, 29.62, 29.60, 29.5, 29.3, 29.1, 28.7, 24.0, 22.7, 19.7 (CH_2_aliph); 13.7 (CH_3_); MS m/z 682.6 ([M-H]^-^, 20%) and 249.5 (M- -433). Anal. calc. for C_38_H_53_NO_8_S (683.89): C, 66.74; H, 7.81; N, 2.05; S, 4.69. Found C, 66.98; H, 7.59; N, 2.35; S, 5.00. 

#### 2.2.8. Synthesis of 2-(2-(methylthio)ethylamino)-3-(dodecylthio)naphthalene-1,4-dione (20)

The solution of 13 (46 mg, 0.19 mmol) and 1-dodecanethiol 18 (70 mg, 0.35 mmol) in ethanol and dichloromethane in the presence of triethylamine (1-2 mL) was allowed to stir at reflux temperature by monitoring the progression of the reaction mixture with TLC. Then, the reaction mixture was extracted with water and CHCl_3_. The organics were dried over sodium sulfate and removed under vacuo; thus, the crude mixture was obtained. The crude mixture was then purified by column chromatography on silica gel (stationary phase) with chloroform (mobil phase) to afford product 20: R_f_ = 0.4 (CHCl_3_); Yield: 60% (51 mg); Dark red viscous oil; IR (ATR): 3305, 2956, 2918, 2849, 1668, 1552, 1498, 1287; ^1^H NMR (CDCl_3_) δ: 8.15 (^1^H, dd, CH_napht_, ^3^J = 7.81 Hz, ^4^J =0.98 Hz); 8.04 (^1^H, dd, CH_napht_, ^3^J = 7.81 Hz, ^4^J = 0.97 Hz); 7.63 (td, ^1^H, CH_napht_, ^3^J = 7.81 Hz, ^4^J = 0.98 Hz); 7.72 (td, ^1^H, CH_napht_, ^3^J = 7.81 Hz, ^4^J = 0.98 Hz); 6.70–6.80 (brs, ^1^H, NH), 4.14 (t, 2H, NH-CH_2_, ^3^J = 6.36 Hz), 2.84 (t, 4H, 2 x CH_2_-S, ^3^J = 6.45 Hz), 2.17 (s, 3H, S-CH_3_), 1.20–1.35 (m, 20H, CH_2_aliph), 0.75–0.95 (m, 3H, CH_3_aliph); ^13^C NMR (CDCl_3_) δ: 183.05, 181.35 (C=O), 134.54, 133.69, 132.10, 130.78, 128.83, 126.53 (C_napht_, CH_napht_), 43.71 (NH-CH_2_); 35.01, 34.67, 31.91, 29.97, 29.58, 29.54, 29.34, 28.92, 22.69 (CH_2_aliph), 15.07 (-S-CH_3_), 14.11 (CH_2_-CH_3_); MS m/z 447.1 ([M]^-^, 100%). Anal. calc. for C_25_H_37_NO_2_S_2_ (447.7): C, 67.07; H, 8.33; N, 3.13; Found C, 67.37; H, 8.10; N, 3.38.

## 3. Results and discussion

Initial investigation began with the reactions of 5 with different 1,4-(benzo/naphtho)quinones (1, 2, and 3) to yield a series of new benzoquinone and naphthoquinone derivatives (6–8, 10) as shown in Scheme. Secondly, 2-bromo-1,4-naphthoquinone 4 reacted with and 2-(methylthio)ethylamine 11 to yield 2-(NH-substituted)-3-bromo-1,4-naphthoquinone 12 and 2-(NH-substituted)-1,4-naphthoquinone 13. The reaction between 13 and 1-dodecanethiol 18 resulted NH,S- substituted naphthoquinone compound 20. In addition, 4 reacted with 14 and 16, respectively, to produce crown-containing 1,4-naphthoquinones 15 and 17. NH,S-substituted- and having crown ether moiety 1,4-naphthoquinone compound 19, was synthesized the reaction between 17 and 1-dodecanethiol 18.

**Scheme:**
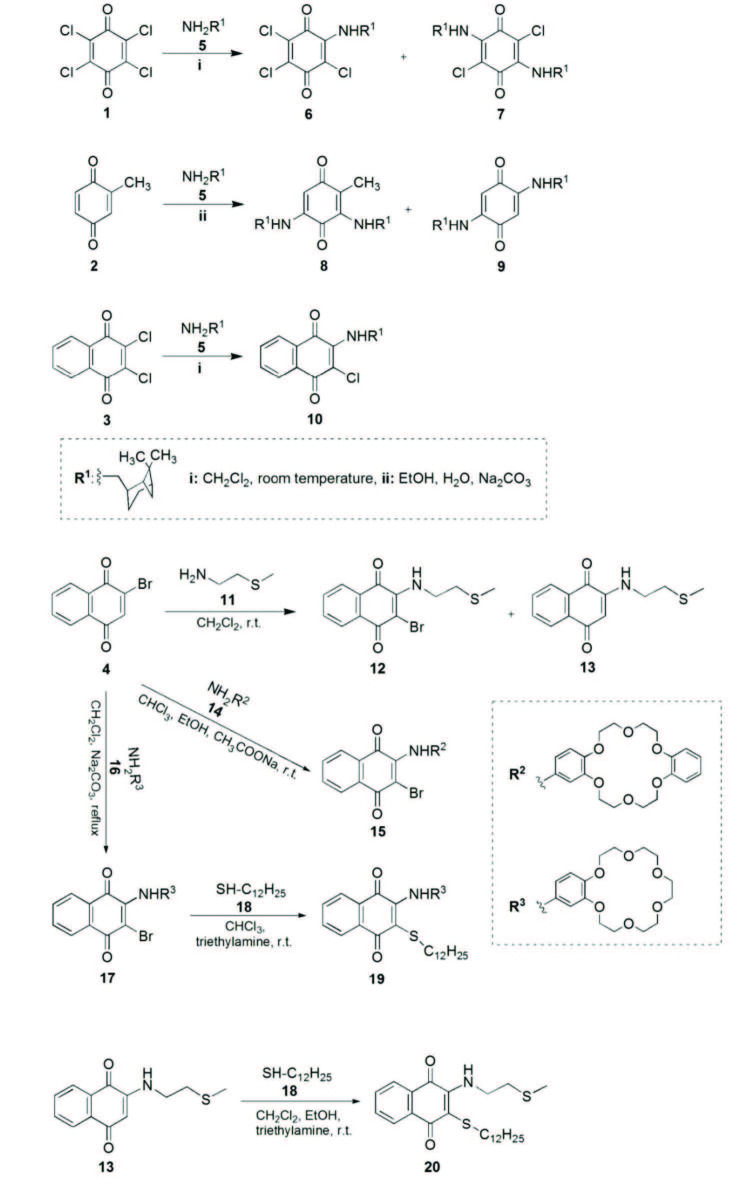
The synthesis of compounds 6-10, 12, 13, 15, 17, 19, and 20.

The reaction between chloranil and primary/secondary amines gives the NH-/N-substituted quinones. Some examples of such reactions have been previously described [51–54]. For example, Singh Gautam BP et al. synthesized and characterized the compound 2,5-dichloro-3,6-bis-(methylamino)-1,4-benzoquinone, which was capable of forming molecular complexes like chloranilic acid [54]. In this work, compounds 6 and 7, having mono-NH-substituted-tri-chloro-1,4-benzoquinone and 2,5-dichloro-3,6-bis(NH-substituted)-1,4-benzoquinone structures, respectively, were synthesized by the reaction of 1:1 molar ratio of p-chloranil 1 with (-)-cis-myrtanylamine 5 in dichloromethane at room temperature. The ^13^C-NMR spectrum of compound 7 shows three symmetric carbon signals at quinone moiety, at 172.12 ppm (C=O), at 145.44 ppm (C-N) and at 99.23 ppm (C-Cl). Moreover, ^1^H-NMR spectrum of 7 showed N-H proton at 7.18 ppm (brs) and other protons at 0.9-4.0 ppm region. Mass spectra of 6 and 7 exhibited m/z [M-H]^-^ = 360.4 and m/z [M+H]^+^ = 479.1, respectively, as expected.

The reactions between methyl-substituted quinones and amines were studied by Cameron et al. [55,56]. For example, o-Xyloquinone with methylamine gave 2-methyl-3,6-bismethylamino-1,4-benzoquinone (39% yield) by displacement of a methyl by an amino-group [56]. Then, Kumanotani et al. carried out the reaction of toluquinone with excess *n*-butylamine [57]. Thus, the results obtained from the study gave the formation of both of 3,6-bis-(*n*-butylamino)-toluquinone (32%) and 2,5-bis(*n*-butylamino)-*p*-benzoquinone (8%, not including methyl group) [57]. Similarly, in the present work, methyl-*p*-benzoquinone 2 was reacted with primary amine 5 in equimolar ratio in EtOH and water in the presence of Na2CO3 to afford 3,5-bis(NH-substituted)-2-methyl-p-benzoquinone 8 (10%) and 2,5-bis(NH-substituted)-p-benzoquinone 9 (78%, not including methyl group). Moreover, compound 9 was synthesized in our previous study [47] but from the reaction between p-benzoquinone and primary amine 5 in equimolar ratio in dichloromethane. While CH_3quinone_ proton and carbon signals of 8 could be observed in ^1^H and ^13^C-NMR spectra at 2.07 ppm and at 10.44 ppm, respectively, in the ^1^H and ^13^C-NMR spectra of 9, the disapperance of CH_3quinone_ signals supported to the formation of 2,5(NH-substituted)-p-benzoquinone structure 9. Moreover, mass spectra of 8 and 9 exhibited peaks at m/z [M+H]^+^ = 425.3 and m/z [M+H]^+^ = 411.3, respectively.

In the literature, there are some reports on the different location of mono- or bis- (NH) groups on the methyl-1,4-quinone moiety, which including 3,5-bis(NH-substituted)-2-methyl-*p*-benzoquinone, 3,6-bis(NH-substituted)-2-methyl-*p*-benzoquinone, 2-(NH-substituted)-6-methyl-1,4-benzoquinone, 2-(NH-substituted)-5-methyl-1,4-benzoquinone derivatives [37,58-60]. In this work, 8 has 3,5-bis(NH-substituted)-2-methyl-p-benzoquinone structure. 

Monosubstitution of the 2,3-dichloro-1,4-naphthoquinone 3 with (-)-cis-myrtanylamine 5 was obtained by using dichloromethane as the solvent to yield compound 10. ^1^H-NMR spectrum of 10 showed two doublet of doublets due to CH_napht_h (8.07, 7.95 ppm) and two doublet of triplets CH_napht_h (7.64 and 7.53 ppm) with proper splitting patterns. In addition, compound 10 displayed signal due to amine (-NH) at 6.03 ppm. 

The reaction of 4 with 11 yielded two new amino-substituted-1,4-naphthoquinones (12 and 13), including bromine and not bromine, respectively. In the ^1^H-NMR spectrum of 13, a singlet appeared at 5.75 ppm, which was assignable to the proton presence of 13 instead of bromine. In addition, in the FTIR spectra of these derivatives (12 and 13) the characteristic bands observed at 1673 and 1664 cm-1 were assignable to the C=O stretching vibrations, respectively.

The reactions of 4 with crown ethers (14 and 16, respectively) were studied and the products 15 and 17 were obtained, respectively. The reaction product 15 had four CH_napht_ peaks at 8.21, 8.13, 7.77, 7.70 and sixteen –O-CH_2_ peaks at 4.13-4.25 (m, 8H), 4.00–4.10 (m, 8H) ppm, in the ^1^H NMR spectrum. In addition, compound 17 exhibited four CH_napht_ peaks at 8.13, 8.04, 7.69, 7.61 and twenty –O-CH_2_ peaks at 4.05–4.13 (m, 4H), 3.82–3.90 (m, 4H), 3.68–3.74 (m, 4H), 3.62–3.68 (m, 8H), in the ^1^H NMR spectrum. 

Compound 17 was reacted with 1-dodecanethiol 18, in the presence of triethylamine, providing both of NH- and SR- substituted-1,4-naphthoquinone 19, which including crown structure. In the proton NMR spectrum of 19, CH_napht_, CH_arom_, and CH_2_crown exhibited signals in a lower field than in the starting compound 17, because of the bonding S-(CH_2_)11-CH_3_ to quinoid structure, instead of bromine. 

To produce NH,SR-substituted-1,4-naphthoquinone derivative 20, 1-dodecanethiol 18 were added a reaction mixture of 13 in solution of dichloromethane and ethanol in the presence of triethylamine. ^1^H NMR spectrum of 20 exhibited methyl proton of 1-dodecanethiolate (–S(CH_2_)11-CH_3_) at 0.89 ppm, methyl proton of –NH-C2H4S-CH_3_ at 2.17 ppm and naphthoquinone protons at 8.15, 8.04, 7.63, and 7.72 ppm, together. In the ^13^C spectra of all synthesized naphthoquinone derivatives (10, 12, 13, 15, 17, 19 and 20), the characteristic signals appeared in the range of 180.0–183.1 and 176.4–181.6 ppm (quinonic carbonyl carbons). Furthermore, it can be clearly seen that the m/z values of these compounds are in ESI mass spectra, as expected. 

## 4. Conclusion

The main goal of this study is to synthesize NH-substituted-1,4-benzo(naphtho)quinones (6-10, 12, 13, 15, 17) starting from different quinones (1, 2, 3, or 4) with amines (-)-cis-myrtanylamine 5 or 4-tert-butylbenzylamine 11. The formation of both of NH- and SR- substituted-1,4-napthoquinones (19, 20) were obtained from NH-substituted-1,4-naphthoquinones 17 and 13 with 1-dodecanethiol 18, respectively. Moreover, compounds 15, 17, and 19 included crown-ether moiety. Medium yields (80% and 60%) were observed for NH-,S-substituted naphthoquinones (19 and 20), whereas lower yields were generally produced for NH-substituted naphthoquinones. New products were verified by elemental analysis, UV-Vis, FTIR, ^1^H-NMR, ^13^C-NMR, and MS-ESI spectroscopy. 
